# Supplementary material for: Factors associated with death in patients with tuberculosis in Brazil: Competing risks analysis
Source: PLoS One. 2020 Oct 8;15(10):e0240090. doi: 10.1371/journal.pone.0240090 (PMC7544107; doi:10.1371/journal.pone.0240090)
Supplement: S1 File — (DOCX) [file pone.0240090.s001.docx]

**S1: Supplementary methods: Procedure linkage**

The linkage was performed in three steps. The first one was conducted in SINAN’s database using a deterministic algorithm for semi automatic linking records, similar to those validated by Pacheco et al. [22] and Oliveira et al. [23], with an adaptation to the STATA statistical software. The first task was pre-processing data to ensure that all variables presented the same format. For names, all letters that were upper case or doubles and had accents and different characters were removed. Suffixes such as Junior and Filho were also removed. We also removed terms that indicated the lack of knowledge about the patient's name or the patient's mother (ignored, unknown).

The second task was the removal of duplicate records in SINAN: (1) exact duplications, which are records belonging to the same individual and that relate to the same episode of illness, reported in the same health unit and (2) transfers, which are records belonging to the same patient and related to the same episode reported in different health facilities., as patient may go through several health units throughout the follow-up, in search of clinical or laboratory diagnosis and common or specialized treatment. Besides, at some point in the follow-up, hospitalization may be required. These transfers between health units can be official or spontaneous.

The second step was the linkage between the databases of SINAN and SIM. We used a probabilistic data linkage procedure using a methodology commonly applied for data encryption coding called Bloom filter [24], using the free software R 3.1.2 and package “PPRL” [25]. For this linkage, the following vital fields were employed: patient's name, mother's name, date of birth, and code of the municipality of residence. For each pair suggested in the linkage step, a score ranging from 8,600 to 10,000 was adopted. Thus, the value of the pairs near the lowest score, established as 8,600, were less likely to be correct pairs, and those close to 10,000 were more likely to be from the same individual. After applying the Bloom filters to the identified pairs, some of them were not from the same person, mainly comprising the score range between 8,600 and 9,200, that is, with lower scores.

Finally, in our third step, only for the groups of records found in the probabilistic data linkage between the SIM and SINAN databases, another deterministic data linkage procedure similar to that used in step one described previously was adopted, with the intention of removing from these groups false-positive for example, records not belonging to the same individual, thus increasing the specificity of the pairs found.

After the records linkage process, three analysis groups were created for the causes of death according to ICD-10 codes: i) probable TB deaths, those that had underlying cause with codes A15 to A19 of ICD-10; ii) TB-related deaths, those deaths in which there was no mention of any of the ICD-10 codes (A15-A19), referring to TB in any line of part 1 of the death certificate; iii) deaths from other causes, those deaths in which there was no mention of TB (codes A15-A19 of ICD-10) in any part of the death certificate.
