# Supplementary material for: Factors associated with death in patients with tuberculosis in Brazil: Competing risks analysis
Source: PLoS One. 2020 Oct 8;15(10):e0240090. doi: 10.1371/journal.pone.0240090 (PMC7544107; doi:10.1371/journal.pone.0240090)
Supplement: Attachment — Submitted filename: Response to Reviewer 17_09_20.docx [file pone.0240090.s004.docx]

**Response to Reviewers**

Author's reply to comments:

Dear Dr. David J Horne, PLOS ONE,

First, I would like to thank you for considering our manuscript for publication in PLOS ONE. We are pleased to send the revised version of the manuscript entitled “***Factors associated with death in patients with tuberculosis in Brazil: survival analysis with competitive risks***” for your consideration for publication in PLOS ONE.

We also thank the reviewers for their meticulous review and appreciate the constructive criticism of our work. We received from the reviewers and hope to point out all comments appropriately. We have made a wide revision and changes to the manuscript, which are highlighted and we also addressed the comments and changes, point to point, below. It was an immense privilege to get such valuable feedback that allowed us to address several gaps in our manuscript.

In this letter, you will find our responses to each question and concern.

Kind regards,

The authors

**Additional Editor Comments (if provided):**

In addition to the comments below, Reviewer 2 had previously requested that reference 1 be updated to the 2019 WHO Global Tuberculosis Report. Although the citation was updated, the language in the introduction was not. You still refer to 2017 deaths from TB -- the 2019 report is on deaths in 2018. Please update the year and the accurate number of deaths for 2018.

**Authors comments**: We appreciate the observation, we have corrected the epidemiological data of the global tuberculosis report for the year 2018.

RESPONSES TO THE **REVIEWER #3**

1) Some terms adopted for presenting the statistical methods need to be revised.

I think “survival analysis with competitive risk” is a rarely used term. “survival regression model” is also strange. It should be Cox regression/Cox proportional hazard model, or in your context, Fine and Gray competing risk regression model. Please go through all statistical terms again and make sure that they are standard terms commonly used in literature.

**Authors comments:** We appreciate your important placement. We carried out an extensive review and correction using the same nomenclatures used by one of the creators of the method (the researcher JP Fine), in a recent manuscript "*Practical recommendations for reporting Fine-Gray model analyzes for competing risk data*", for more details: https: / /pubmed.ncbi.nlm.nih.gov/28913837/

2) Besides, expressions such as “we believe”, “databases are fed continuously” should not be in academic writing.

**Authors comments**: Thank you for your observation and we have corrected these expressions in the manuscript.

3) Regarding my previous comments about a more detailed description of the data source, the sample representativeness is still not entirely clear to me. I don’t think “good coverage throughout the country” is precise enough. As this is critical for evaluating the external validity of the results, please try to add more accurate information.

**Authors comments**: We appreciate your important placement. More information was included in the aforementioned manuscript that describes the data source used to link the records, reporting their potential and also limitations of use in Brazil.

4) Regarding the response about how missing values were handled. I think using complete cases is not a big issue. The important thing is to report the procedure - how the final sample size was arrived at (see the STROBE checklist). Even if you did not impute the data (which sometimes, indeed, may create another set of problems), the number of participants involved in each exclusion step needs to be reported (a flow chart is helpful, particularly for cohort study using electronic health records).

**Authors comments:** We are grateful for the reviewer's concern and when re-evaluating the figure of the linkage process presented, it was confusing, as we mixed reclassification information of "treatment status" and exclusion criteria for observations (duplicate observations, inconsistencies in dates, among others). We redid the flowchart only with the information on the exclusions of the observations according to the exclusion criteria of the study cohort.
